# 3D Mitochondria Shape Library for Optical Microscopy (3DMSL): A multimodal dataset for deep learning based mitochondrial analysis

**DOI:** 10.1016/j.dib.2026.112507

**Published:** 2026-01-29

**Authors:** Abhinanda R. Punnakkal, Suyog S. Jadhav, Aaron V. Celeste, Alexander Horsch, Krishna Agarwal, Dilip K. Prasad

**Affiliations:** a Department of Computer Science, UiT The Arctic University of Norway, Tromsø, Norway; b Department of Physics and Technology, UiT The Arctic University of Norway, Tromsø, Norway

**Keywords:** Synthetic dataset, Deep learning, 3D shape modelling, Mitochondria analysis, 3D reconstruction

## Abstract

With the increasing development of deep learning solutions for fluorescence microscopy image analysis, there is a growing demand for annotated ground truth datasets to train supervised methods. However, obtaining these annotations is a laborious and expensive endeavor. To address this problem for microscope analysis of cell organelles, we release 3DMSL, a database of 3D shapes of mitochondria. 3DMSL utilizes high-resolution Electron Microscopy data as the source for creating the extensive database. Utilizing a physics-based simulator, 3DMSL enables the creation of large fluorescence microscope image datasets with 3D ground truths, which can be used to train deep learning models for various applications, including segmentation, 3D reconstruction from images and stacks, creation of time-lapse videos with 3D ground truth, and microscope-to-microscope translation. 3DMSL contains >27k instances of diverse mitochondria shapes in different 3D shape representation formats of meshes, point clouds and implicit shapes.

Specifications Table

**Table:** 

Subject	Computer Sciences
Specific subject area	Simulated optical microscope data of mitochondria with 3D ground truth shape in various data forms, aimed at creating training data for deep learning models.
Type of data	Image, mesh, point clouds, and implicit shapes for 27,272 mitochondria shapes.
Data collection	The data is created by sourcing segmentations of high-resolution Electron Microscope images as ground truth shapes.
Data source location	UiT the Arctic University of Norway
Data accessibility	Repository name: DataverseNo Data identification number: https://doi.org/10.18710/JX6JXF Direct URL to data: https://doi.org/10.18710/JX6JXF
Related research article	None

## 1 Value of the Data

• The main motivation for releasing 3DMSL is to enable the **creation of large training datasets of microscope images with 3D ground truth labels**. The dataset contains 27,272 instances of 3D mitochondrial shapes extracted from high-resolution Electron microscope images that provide 3D ground truth shapes for training deep learning models for optical microscope images.
• By using real 3D shapes of mitochondria, the data is representative of the **true shape distribution of mitochondria and contains the different shape classes** of mitochondria identified in literature: dots, rods, donuts, mega, nano, and networks.
• Integrating this accurate representation of shape with a physics-based microscope simulator, we provide **paired microscope images and 3D ground truth shapes** that are ideal for training deep learning models for many applications of microscope analysis.
• We **provide multimodal data for 3D mitochondria shapes, including meshes, point clouds, and implicit representations**. We also include **3 sets of microscope images** varying in their types and configuration (2 Confocal microscopes and 1 epi‑fluorescence microscope).
• Potential **applications** include style and perspective transfer across microscopes, creating 3D microscope image stacks, training data for segmentation, 3D reconstruction, and generating synthetic dynamic behaviour datasets for mitochondria.

## 2 Background

3D mitochondrial shape modelling is vital for understanding their role in energy production and diseases like neurodegenerative, cardiovascular, and metabolic disorders. Structural abnormalities indicate dysfunction, which can be quantified via automated analysis of fluorescence microscope (FM) images. This analysis aids in the quantification of dysfunction [1,2], assessing drug efficacy [3], and understanding dynamic processes like fission and fusion. However, FM images have poor spatial resolution, leading to loss of 3D structural details and misinterpretations (d), such as mistaking tubular mitochondria for ring shapes [4]. Different tools for mitochondria analysis based on image processing [[5], [6], [7]], and deep learning [1,[8], [9], [10]] exist, but annotated datasets are scarce due to tediousness and subjective variability [1].

Correlated Light-Electron Microscopy (CLEM) is one way to obtain 3D ground truth for FL. However, it is time-consuming and requires expensive equipment. Synthetic data generation has emerged, but struggles to capture the complex geometry of mitochondria [5] and the diversity of mitochondrial shapes [2]. Thus, 3DMSL addresses the needs for a realistic synthetic dataset of mitochondrial shape with ground-truth labels. Using 27 K Electron Microscope (EM)-derived shapes and a physics-based FM simulator, it enables training deep learning models to accurately model 3D mitochondrial shapes.

## 3 Data Description

The entire dataset of 3DMSL is made available through DataverseNo in the URL https://doi.org/10.18710/JX6JXF and can be cited as [11]. 3DMSL has a total of 27,272 instances of mitochondria shapes divided into 10 zip files. The list of files in 3DMSL data repository is

1) 00_ReadMe.txt
2) 1_1_2729.zip
3) 2_2729_5457.zip
4) 3_5457_8185.zip
5) 4_8185_10,913.zip
6) 5_10,913_13,641.zip
7) 6_13,641_16,369.zip
8) 7_16,369_19,097.zip
9) 8_19,097_21,825.zip
10) 9_21,825_24,553.zip
11) 10_24,553_27,272.zip
12) meta_locations.csv

**Fig. 1:**
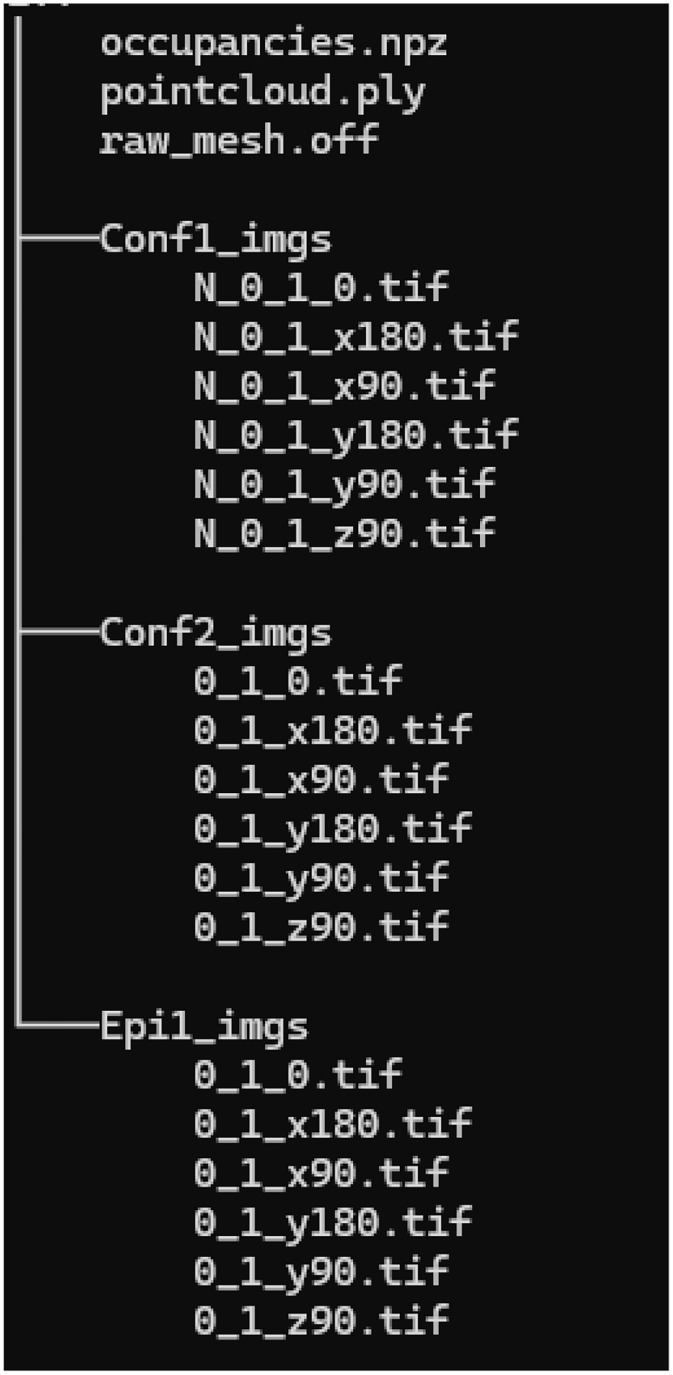
Screenshot of the folder structure and contents of one mitochondria instance in the dataset.

**Fig. 2:**
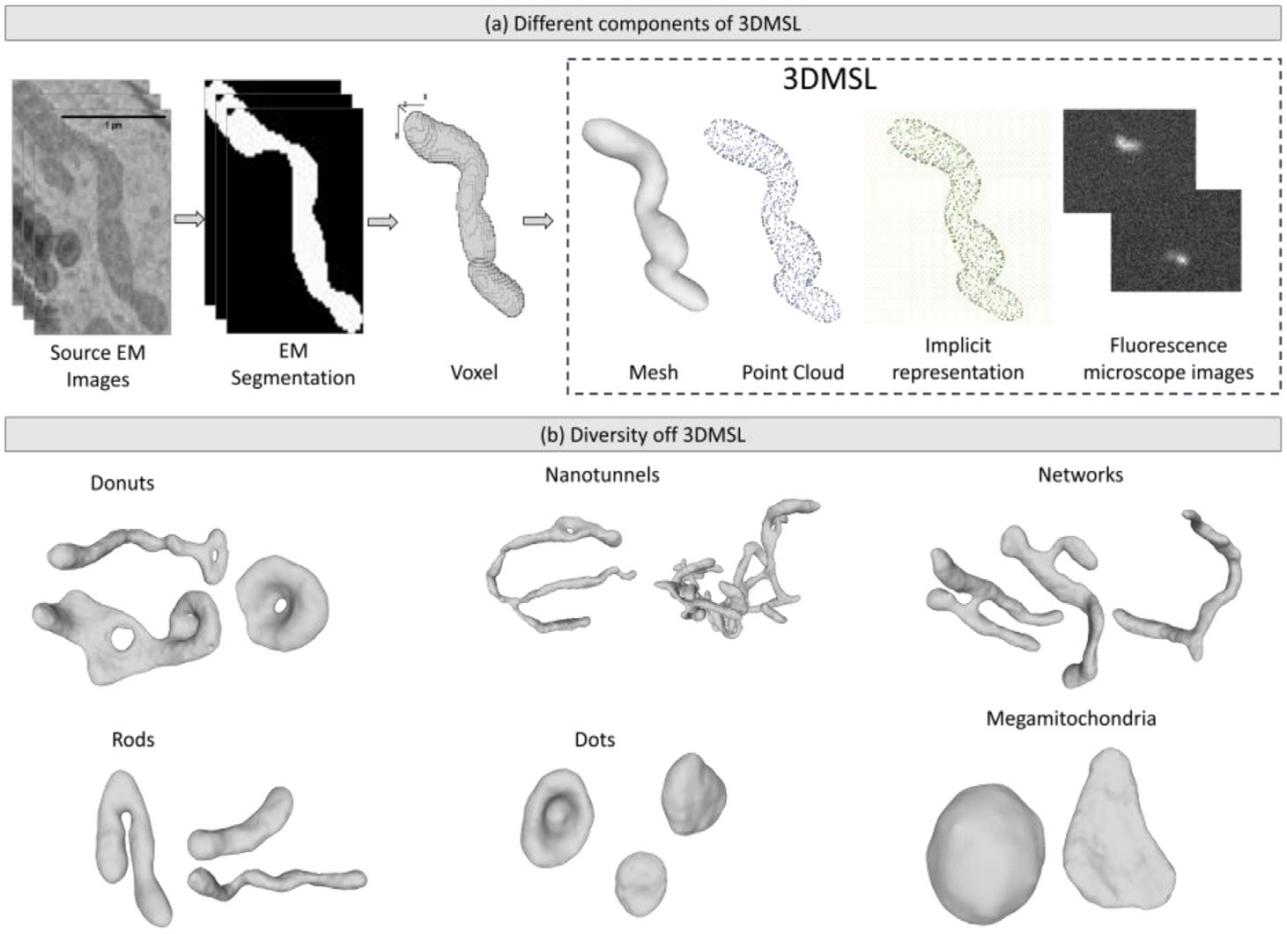
(a) 3DMSL includes different shape representations and fluorescence microscopy images of each mitochondrion instance (b)Diversity in shapes of mitochondria instances present in 3DMSL. 3DMSL contains the different shapes of mitochondria identified in literature: dots, rods, donuts, mega, nano and networks.

Each zip file contains ∼2728 instances. Each instance is presented in three 3D shape formats (Fig. 2(a)) for each mitochondrion: a 3D volume with surface mesh (raw_mesh.off); a 3D point cloud with normals, sampled from the 3D volume (pointcloud.ply); and an occupancy array (occupancies.npy). Additionally, the dataset also contains simulated microscopy images for the three microscope configurations mentioned in the paper (two confocal configurations: Conf1 and Conf2, and 1 Epifluorescent configuration: Epi1). There are 6 total simulated images for each microscope configuration of an instance, computed from 6 different angles of viewing. A complete list of the files contained within the different instance folders is provided in the file ‘00_ReadMe.txt’ and shown in Fig. 1.

All files related to an instance are zipped (losslessly) together, for efficient uploads and downloads, and the start index is 1. The naming convention of the zip files is "{zip_number}_{start_index_of_instance}_{end_index_of_instance}", where

• {zip_number} - indicates serial number of the zip folders.
• {start_index_of_instance} - indicates the start index of the instances in the zip
• {end_index_of_instance} - indicates the end index+1 of the instance in the zip

For example, "1_1_2729.zip" is the first zip folder of 3DMSL and contains data instances from 1 to 2728. A summary of all the different file type sof each instance, descripption and approximate file sizes are provided in Table 1.

**Table 1:** Summary table of all the file types and their contents for each instance of 3DMSL.

#	Name	Description	Filetype (Extension)	File Size range	Count per instance
1	Occupancies.npz	The file contains the occupancy query points and the ground truth for the points.	Numpy file (npz)	Constant, 600KB	1
2	Pointcloud.ply	The file containing the 3D point cloud format of the instance.	Polygon file format (ply)	Constant,5.5 MB	1
3	raw_mesh.off	The file containing the 3D mesh of the instance.	Object File Format (.off)	Varying, 123KB- 21MB	1
4	Conf1_imgs/N_0_{instance_number}*.tif	Synthetic microscope images of the instance for microscope setting Conf2	Tagged Image File Format (.tif)	Varying, 32KB-37KB	6
5	Conf2_imgs/0_{instance_number}*.tif	Synthetic microscope images of the instance for microscope setting Conf1	Tagged Image File Format (.tif)	Varying, 32KB-36KB	6
6	Epi1_imgs/0_{instance_number}*.tif	Synthetic microscope images of the instance for microscope setting Epi1	Tagged Image File Format (.tif)	Varying, 32KB-36KB	6

The instances in this dataset were extracted from the ‘1857 Obsese Climp63 Liver dataset’ [12]. We include a file (meta_locations.csv) containing the locations of the extracted mitochondria in the original dataset. The metadata includes the coordinates of the bounding box of the mitochondrion in the original dataset, the number of voxels contained in the bounding box, and the centroid coordinates of the bounding box.

For easy selection of different shapes available, we coarsely classify the 27k instances of 3DMSL into shape classes. We refer to the literature [13] for classification of 3DMSL into dots, rods, networks, nano-tunnels, mega mitochondria, and donuts. We show examples of these classes in Fig. 2(b).

We also provide a subset of the dataset for easy download and visualization of instances of 3DMSL through the url: https://figshare.com/s/7e4037d280ad61c5af05.

## 4 Experimental Design, Materials and Methods

### 4.1 Dataset generation

**Fig. 3:**
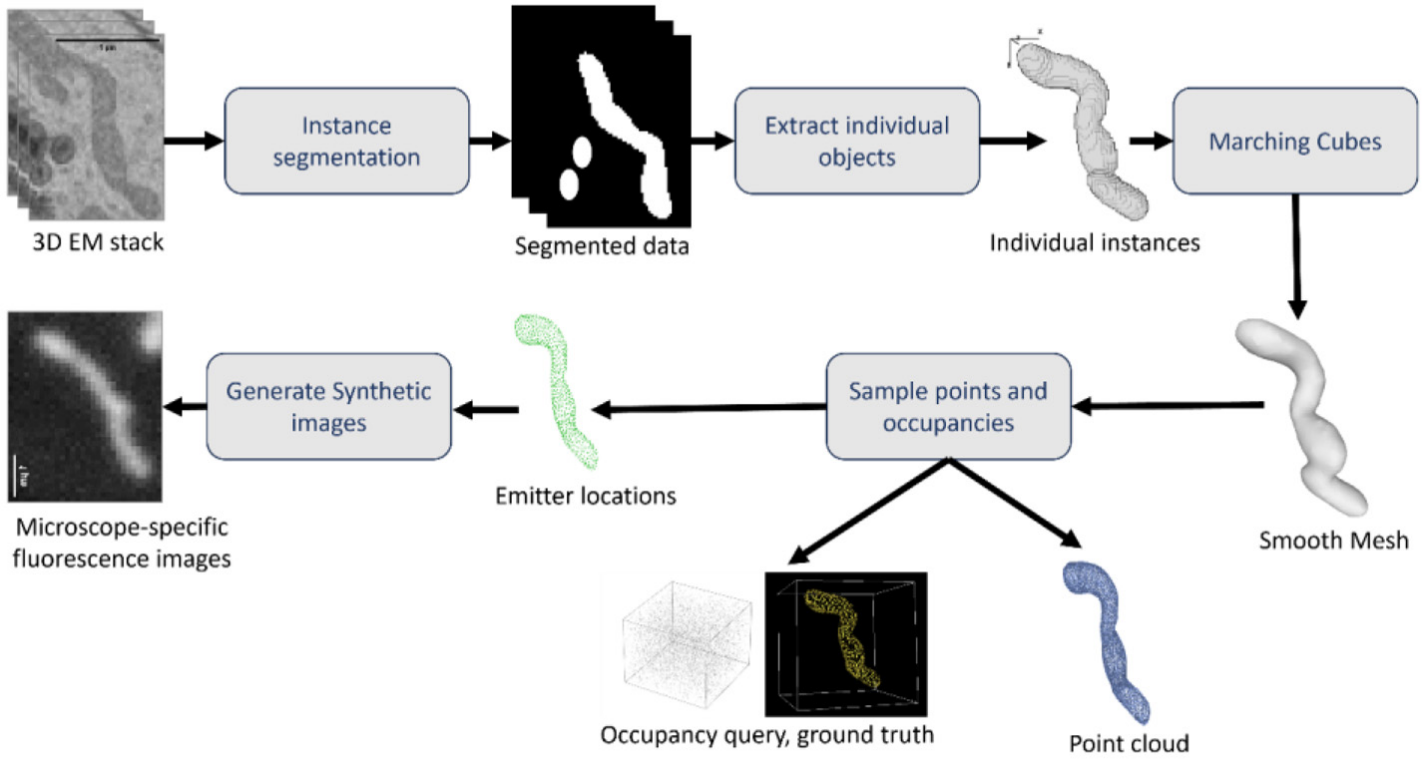
Flowchart of the dataset creation steps.

This subsection describes the source and protocol followed to create the different forms of 3D shape representation available in 3DMSL. A Concise flowchart of the dataset creation steps is shown in Fig. 3. A table summarizing the values and description of the parameters used in the different steps of dataset creation is given in Table 3.

#### 4.1.1 High resolution data source

Our dataset is sourced from publicly available raw EM data of liver cells published by Parlakgül, G. et al. [12]. Other EM sources like [14] either have a lower number of shape instances than or are computationally demanding. Parlakgül, G. et al. [12] consists of 4 sets of 3D EM data that are obtained using a Focused Ion Beam Scanning Electron Microscopy (FIB-SEM). The 3DMSL source dataset is of liver of obese mice, where Climp63, a protein associated with the endoplasmic reticulum (ER), is overexpressed. The liver, a critical organ for metabolic processes such as lipid metabolism, glucose regulation, and insulin signaling, is often disrupted in obesity. The data was acquired as part of the study to investigate obesity-related liver dysfunction, including altered ER morphology, mitochondrial dysfunction, lipid droplet accumulation (indicative of steatosis or fatty liver disease), and changes in cell membrane integrity. The animal model used is leptin-deficient B6.Cg-Lepob/J (ob/ob) male mice, genetically predisposed to obesity due to leptin deficiency. Interventions include adenovirus-mediated overexpression of proteins related to ER structure and function, such as Climp63, which stabilizes ER sheets. Adenovirus was administered to obese mice at a dose of 1.1 × 10⁹ infectious units (IFU). The mice were maintained on a 12-hour light/dark cycle with free access to water and a standard chow diet, and experiments were conducted between 8 and 12 weeks of age. This dataset provides insights into the cellular and subcellular mechanisms underlying obesity-related liver dysfunction and metabolic disorders. The segmentation labels using the 3dEMtrace platform of ariadne.ai^1^ are also available. These images of sub-cellular structures are at an isotropic resolution of 8 nm. We select one out of the 4 available data-label pairs for creating 3DMSL.

#### 4.1.2 3D shape extraction protocol

We briefly describe the steps used by Parlakgül, G. et al., to obtain the segmentation masks of mitochondria that we use as our source dataset. The segmentation of mitochondria provided by Parlakgül, G. et al. is obtained using a semi-automated deep learning-based approach [12]. A 3D U-net-based architecture was used and trained using a subset of the data that is manually annotated with mitochondria segmentation labels. The model is then fine-tuned to generate segmentation masks for the entire data volume. The masks were verified quantitatively and corrected [12] by experts.

These verified segmented EM images from [12] is the source of our data. These segmented images are resampled at 24 nm for computational efficiency to extract the 3D shape of individual mitochondria by applying the connected components algorithm [15] (Requires 1.8 TB RAM at 24 nm, which was run on Oracle cloud compute). To counteract the merging effects of downsampling from 8 nm to 24 nm, the 3D segmented data is first eroded using a spherical structuring element of radius 3.5 voxels. Thereafter, individual components that correspond to one mitochondrion each are extracted and then dilated using the same structuring element as used during erosion. In this manner, we formed a total of 27,272 instances of 3D mitochondria shapes, which form the voxel representation released by 3DMSL. These instances are visualized to check for shape inconsistencies.

The protocol used for creating these different data forms of 3DMSL is discussed in the methods section. We adapt steps from [16] for creating the various 3D forms of the instances. 3DMSL consists of the following formats:

**Mesh**: Meshes of individual mitochondria are extracted using the marching cubes algorithm that extracts the 0-isosurface of the object. A Gaussian filter is applied to smooth the surface of these meshes.

**Pointcloud**: One use case for our data is mesh reconstruction from point clouds that are contaminated with noise. To achieve this objective, we select a subset of 300 points from the surface of each mesh within the 3DMSL dataset. Subsequently, we introduce noise to these point clouds by perturbing them with a Gaussian distribution that has a zero mean and a standard deviation of 0.05.

**Implicit Representation**: We include implicit representations in 3DMSL because of their advantages when used in deep learning networks. Occupancy is an implicit 3D shape representation that represents 3D surfaces as the continuous decision boundary of a deep neural network classifier. To create this representation, the meshes are prepared for the occupancy networks as follows. First, each mesh is normalized to fit into a unit cube of dimension 1. No stretching or skew is introduced, and the operation is equivalent to performing isotropic scaling of Cartesian coordinates. Then, 10,000 points are sampled uniformly in the unit cube, and their occupancy values are computed. This pair of query points and occupancy values form the occupancy representation [16].

**Fluorescence Microscope images**: We also release synthetic microscope images corresponding to these instances for different fluorescence microscope types and configurations.

We generate large training datasets for three types of fluorescence microscope images: an epifluorescence microscope (Epi1) and two confocal microscopes (Con1 and Con2). The exact parameters used for Epi1, Con1, and Con2 are described in Table 2. These differ in their optical designs, digital resolutions, and image qualities. Epifluorescence microscopes are high-throughput microscopes, but with poor z-sectioning (DOF ∼500 nm) and poor lateral resolution (∼250 nm) while confocal microscopes are low-throughput but have superior z-sectioning (DOF ∼250 nm) and superior lateral resolution (∼180 nm).

**Table 2:** Microscope Settings used for various experiments.

**Microscope**	Con1	Epi1	Conf2
Type	Confocal	Epifluorescence	Confocal
Emission wavelength	600 nm	688 nm	488nm
Pixel Size	70 nm	109 nm	48 nm
Numerical Aperture	1.4	1.42	1.4
Magnification	63	60	63
Optical Resolution	152 nm	245 nm	124 nm

**Table 3:** Parameter table summarizing values used for parameters in different steps of dataset creation.

Parameter	Step Used	Description	Value (Unit)
Downsampling factor	Extract individual objects	The factor used to downsample the segmented image stack for computational efficiency of the connected components algorithm.	3
Erosion diameter	Extract individual objects	The diameter of the kernel used for erosion before running the connected components algorithm	3.5 (pixels)
Dilation diameter	Extract individual objects	The diameter of the kernel used for dilation after running the connected components algorithm	3.5 (pixels)
Point cloud count	Sample point cloud and occupancies	Number of points sampled on the mesh surface for the pointcloud representation.	300
Point cloud noise std	Sample point cloud and occupancies	Standard deviation of the gaussian distribution used to perturb the point cloud values to impart noise in the point cloud representation.	0.05
Occupancy point count	Sample point cloud and occupancies	Number of points sampled in the unit cube containing the normalized object for creating the implicit representation.	10,000
Fluorescent molecule density	Generate synthetic images	The density of fluorophore molecules which is the sampling density for emitter locations on mesh instances.	30 points /µm^2^

The fluorescence microscopy images are created of individual mitochondria using two major steps, namely, simulating the fluorescence labelling by computing fluorescence molecule distribution on the shape, and generating the fluorescence image from the fluorescence molecule distribution.

We simulate fluorescence labelling by sampling points on the mesh surface and using them as the fluorescence molecules’ binding locations. The number of points is determined by the density of fluorescence molecules that the mitochondrion is expected to be labelled with. For example, if an outer membrane fluorescent label is used and the expected surface density of fluorescent molecules is 30 molecules/µm^2^, then a sampling density of ∼30 points /µm^2^ is used. We create different perspectives of the individual objects by rotating the mesh object along different axes.

**Fig. 4:**
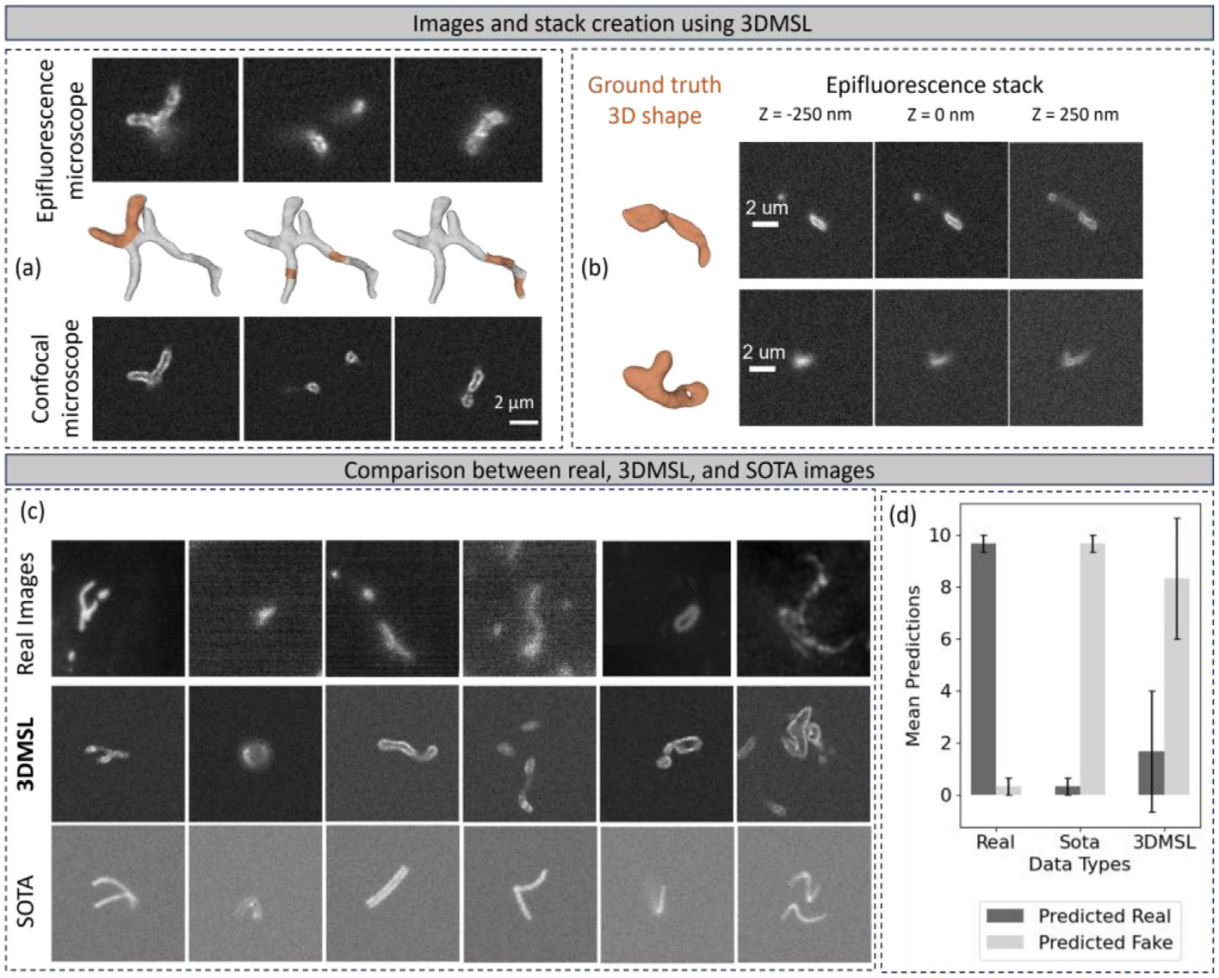
(a) Illustrations of fluorescence microscopy images generated from different rotations of a mitochondrial network in 3DMSL. The regions in orange on the 3D shape denote the regions that fall in the focal plane. (b) Illustrations of fluorescence microscopy image stacks generated by scanning the focal plane of a mitochondrial network in 3DMSL. (c) Example images for comparison between real, 3DMSL and SOTA used in the human evaluation experiment. (d) Result of the human evaluation experiment.

Generating the fluorescence image from the sampled fluorescent molecule locations is carried out by applying a computationally efficient version of the Point Spread Function (PSF) [17] on the sampled fluorescent molecule locations. We used the microscope simulators publicly released by [2]. Noise in the images is simulated using the noise model presented in the same source. We present in Fig. 4(a), examples of fluorescence microscopy images created from a complex mitochondrial shape present in a 3D EM image. The simulated images show mitochondria at different perspectives for different microscope settings.

**Metadata** We also release the metadata of the individual instances in the context of the overall EM volume in 3DMSL. This includes voxel count, centroid location, and the bounds in the x,y, and z axes for each mitochondria instance. It also includes a computed class of the individual shapes based on different types of shape classes identified in the past [13]. These classes are dots, rods, networks, nano-tunnels, mega mitochondria, and donuts. Skeletonization is applied to the 3D shapes, and classification is determined by the topology and length of the skeleton. Any skeleton with loops in topology are classified as donuts. All instances are classified based on their skeleton lengths as well. Any mitochondria <1 µm skeleton length is classified as dots, greater than 30µ m as nano-tunnels, skeleton length greater than 10µm and has a volume greater than 1µm^3^ as mega mitochondria, and the rest as rods if there are no branches and networks otherwise.

### 4.2 Dataset validation

The synthetic microscope datasets created by following the steps described in the section dataset creation, can be used for a variety of supervised deep-learning applications. Fig. 4(a) shows an example of microscope images created using an instance of 3DMSL for different microscope types. The same instance is rotated (along the rows), to get fluorescence microscopy images for different perspectives of the object. This implies that we can perform high-quality data augmentation. Even though this example shows data augmentation for 2D images, the concept easily generalizes to z-stack data augmentation (not included in the dataset, the code used to create the example z-stack is provided in the associated GitHub page). To generate a z-stack of microscope images, we extend the steps described in the methods section and scan the focal plane of the microscope with respect to the instance. Fig. 4(b) illustrates examples of 3D epifluorescence microscope image stacks created from the instances of 3DMSL with an axial step size of 250 nm.

Fig. 4(c) shows a comparison between real microscope image and synthetic microscope images created using 3DMSL and using cylindrical geometry as used in Sekh. et al. [2]. The synthetic images created using 3DMSL are more realistic than the SOTA [2] synthetic image creator. We compute the Fréchet inception distance (FID) [18] between a set of 30 real images, a set of synthetic images generated using 3DMSL protocol, and the set of synthetic images from SOTA, which were used in their quantitative human evaluation experiment. The FID score for our proposed dataset, in comparison to real images, is 101.689, which is significantly lower than the FID score of the SOTA dataset FID score of 146.229. This indicates a higher similarity between our dataset and the real images.

We conduct a preliminary evaluation to assess the fidelity of microscope datasets created using 3DMSL by conducting a human evaluation experiment. This is done by showing participants a mix of real microscope images, images created using 3DMSL, and images created using the SOTA [2], and the participants are asked to judge if the image is real/fake. Compared to the SOTA, our shapes are far from perfectly smooth cylinders as they include all the bumps, lumps, and organic morphology quirks of the shapes. Fig. 4(d) shows the results of this experiment. We note that the number of participants in this experiment is 9, which is small to obtain statistically significant results. The results indicate that our images are rated more realistic than SOTA. However, the accuracy of the participants is high due to the difference in the noise present in 3DMSL and real images, as commented by participants.

**Fig. 5:**
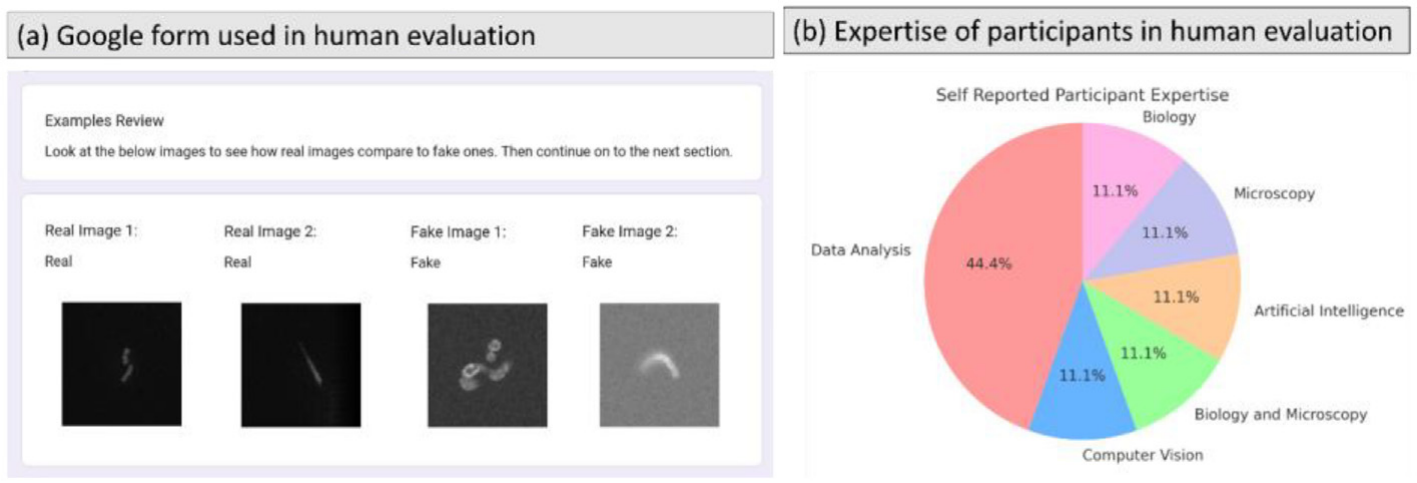
(a) Google form used in the human evaluation experiment to test realism of the images created using 3DMSL. The figure shows examples shown to the participants. (b) The self-reported areas of expertise of our Google Forms study participants.

The details of the study are as follows. The study recruited 9 experts whose self-reported areas of expertise are shown in Fig. 5(b). These were participants already familiar with mitochondria images and working on their expertise using mitochondrial images. The study asked participants whether an image looked like it was taken through a microscope or whether it depicts "a fake scene which is computer-generated using a 3D simulation engine". The images in our study were a mix of SOTA [2], 3DMSL images, and real microscopy images. Participants were shown examples of 2 labeled real images and 2 labeled simulated ones, as shown Fig. 5(a).

Each participant assessed a set of 30 images containing 10 SOTA, 10 real, and 10 3DMSL images. Each image is assessed by 3 participants. All members are exposed to and actively working on fluorescence microscopy of mitochondria data. The results of this experiment are presented in Fig. 4(d). These results indicate that our images are more realistic than SOTA. However, they are not statistically relevant as the accuracy of the participants remains high, as the synthetic images are distinguishable from real images. This is attributed to the difference in the noise present in 3DMSL and real images, as commented by participants. So, for user wanting to use the microscope images might also consider further noise addition to match the noise levels of their experimental real images.

The results of T-Test Results are as follows: Real vs SOTA (*t* = 36.36, *p* = 0.0), Real vs 3DMSL (*t* = 15.51, *p* = 0.0000000046), SOTA vs 3DMSL (*t* = −2.60, *p* = 0.0240061769), which indicate a higher p-value between real data and 3DMSL than real and SOTA.

### 4.3 Application - training 2D segmentation of mitochondria using 3DMSL improves IoU and topological similarity

We benchmark the effectiveness of our data using the 2D segmentation model used in PhysSeg [1] for mitochondrial image segmentation in Table 4. To test on real data, we use the manually annotated dataset from PhysSeg [1] consisting of 279 training images and 144 test images.

**Table 4:** A quantitative comparison of the segmentation methods tested on real images. The averaged values over all the test images and the standard deviation are shown.

**Method**	Betti No.s Error ↓	**Dice Score** ↑	**IoU** ↑
PhySeg	3.551 ± ^2.789^	0.859 ± ^0.057^	0.757 ± ^0.080^
**3DMSL**	**3.430 ±**^3.121^	**0.864 ±**^0.075^	**0.767 ±**^0.088^

**Fig. 6:**
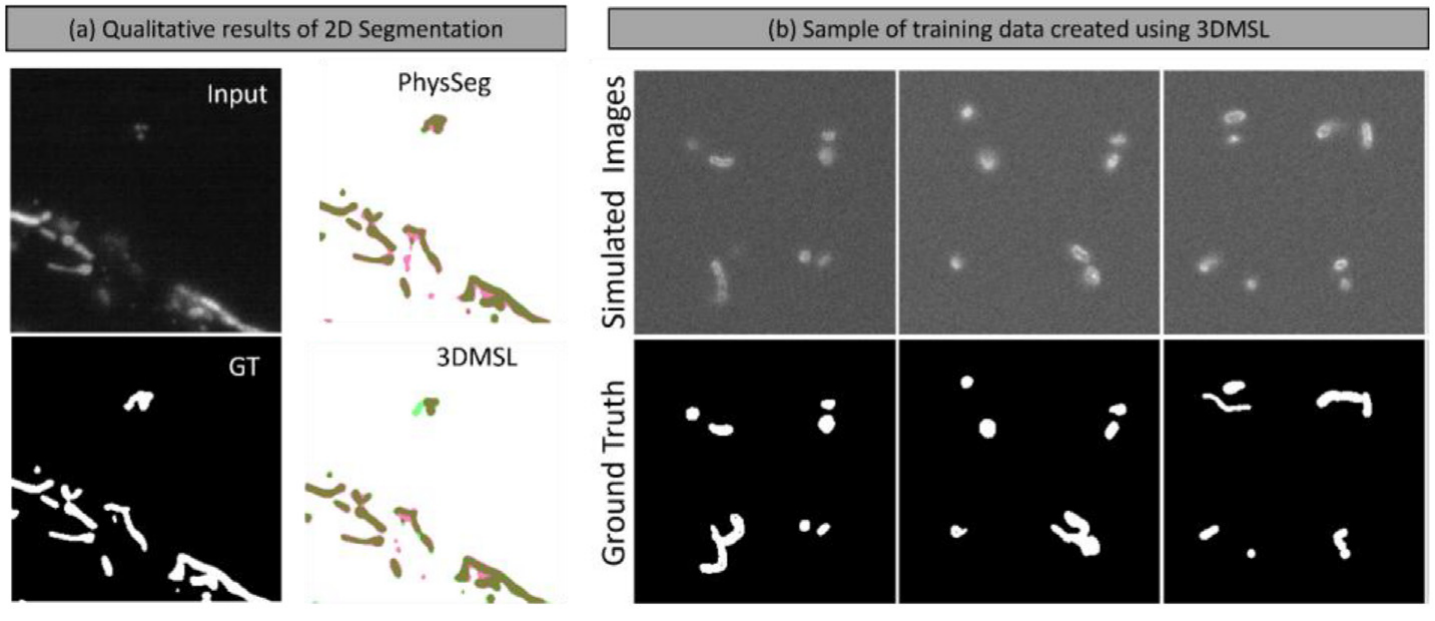
(a) Qualitative results comparing the performance of 2D segmentation on real images. Green regions in the segmentations are false negatives (missed pixels), pink regions are false positives, and other white or dark regions are correct detections. (b) Examples of our simulated training dataset created using 3DMSL data for 2D Segmentation.

To demonstrate the utility of 3DMSL for the task of 2D segmentation, we create a dataset of optical microscope images and their 2D ground truth segmentation masks created from 3DMSL for the problem of 2D segmentation. We create a simulated dataset of 7000 images matching the parameters of the real microscope data from an epifluorescence microscope. We show example images of this dataset in Fig. 6(b). Similar to PhysSeg, we then use this synthetic data to train a segmentation model with an EfficientNet backbone. Segmentation metrics of dice score and F1 score are computed along with Betti error. Betti error allows us to verify how accurately the general topology of the segmented object is being preserved by the given segmentation method. We used Betti error function implemented in [19], which allows matching two geometries in terms of the number of components and the number of holes, with the lower Betti error indicating a better match.

Both PhySeg and 3DMSL are pre-trained on synthetic datasets, of which the model trained using 3DMSL has the best segmentation results as indicated by the highest dice and IoU scores. (Note: PhySeg and 3DMSL entries are the performance of the same segmentation model trained on different synthetic datasets. PhySeg and our model do not differ in architecture, but only in the data that is trained on. Qualitative examples demonstrating the performance of the two dataset is provided in Fig. 6(a). We see that the model trained using 3DMSL has the best performance in both segmentation metrics and topological metric." => "From Table 4, we see that the model trained using 3DMSL has the best performance in both segmentation metrics and topological metric.

## Limitations

At present 3DMSL uses only one source of data, from mouse liver cells. Mitochondria in tissues and other cell types like heart cells and neurons are different from commonly observed types and thus would restrict the use of 3DMSL for these samples. The quality of 3D EM segmentation is also a crucial factor in the overall quality of the dataset. Our data is good candidate for simulations at the resolution of light microscopy (≈200 nm). However, we advise caution if using for super-resolution microscopes of resolution <50 nm. Our source data is at a resolution of 8 nm and we perform connected component analysis at a resolution of 24 nm. The details of shape in 3DMSL depend on the accuracy of EM segmentations. These segmentation masks were obtained by semi-automated EM segmentations and verified by human experts by the providers of the dataset [12]. However, for instances that are separated by just several nms, we see that our dataset creation pipeline will create merged objects. This is considered a low-risk problem when working with fluorescence images, but if working in the super-resolution regime, more work is required to separate falsely merged instances.

## Ethics Statement

After consulting the data protection officer at UiT The Arctic University of Norway, a notification to Sikt (*Norwegian Agency for Shared Services in Education and Research* [Reference number: 912,829]) was submitted for assessment of the human evaluation experiment in the dataset validation section. Sikt assessment concluded that as the collected data has now been all anonymized, and all contact information deleted shortly, and only aggregated data in the form of pie charts and tables now exists, no further notification of Data Protection Authority is required.

## CRediT Author Statement

A.R.P created the dataset. A.R.P and S.S.J created the code accompanying the dataset and prepared the dataset for publication. A.V.C validated the dataset. K. A, D.K.P and A.H contributed to discussions, analyzed the dataset, and provided supervision. A.R.P and K.A drafted the manuscript. K.A, A. H and D.K.P acquired funding for the project. All authors reviewed the manuscript.

## Data Availability

Dataverse3D Mitochondria Shape Library for Optical Microscopy (3DMSL) (Original data).

## Footnotes

1 https://ariadne.ai/.

## References

[1] Sekh A.A. (Dec. 2021). Physics-based machine learning for subcellular segmentation in living cells. Nat. Mach. Intell..

[2] A.A. Sekh, “skarifahmed/physeg: physics based machine learning for sub-cellular segmentation in living cells,” Jun. 2021, doi: 10.5281/ZENODO.5017066.

[3] Godtliebsen G. (Oct. 2023). High-resolution visualization and assessment of basal and OXPHOS-induced mitophagy in H9c2 cardiomyoblasts. Autophagy.

[4] Miyazono Y., Hirashima S., Ishihara N., Kusukawa J., Nakamura K.I., Ohta K. (Jan. 2018). Uncoupled mitochondria quickly shorten along their long axis to form indented spheroids, instead of rings, in a fission-independent manner. Sci. Reports.

[5] Viana M.P., Lim S., Rafelski S.M. (2015). Quantifying mitochondrial content in living cells. Methods Cell Biol..

[6] Lefebvre A.E.Y.T., Ma D., Kessenbrock K., Lawson D.A., Digman M.A. (Aug. 2021). Automated segmentation and tracking of mitochondria in live-cell time-lapse images. Nat. Methods.

[7] Lefebvre A.E.Y.T. (Feb. 2025). Nellie: automated organelle segmentation, tracking and hierarchical feature extraction in 2D/3D live-cell microscopy. Nat. Methods.

[8] Fischer C.A. (Oct. 2020). MitoSegNet: easy-to-use deep learning segmentation for analyzing mitochondrial morphology. iScience.

[9] Ding Y. (Jan. 2025). Mitochondrial segmentation and function prediction in live-cell images with deep learning. Nat. Commun..

[10] Punnakkal A.R. (2023). Analyzing mitochondrial morphology through simulation supervised learning. JoVE.

[11] A.R. Punnakkal, S.S. Jadhav, A.V. Celeste, A. Horsch, K. Agarwal, and D.K. Prasad, “3DMSL - 3D Mitochondria shape library for optical microscopy.” DataverseNO, doi:10.18710/JX6JXF. 10.1016/j.dib.2026.112507 PMC12907005 41704521

[12] Parlakgül G. (Mar. 2022). Regulation of liver subcellular architecture controls metabolic homeostasis. Nat.

[13] Jenkins B.C. (Apr. 2024). Mitochondria in disease: changes in shapes and dynamics. Trends Biochem. Sci..

[14] Conrad R., Narayan K. (Jan. 2023). Instance segmentation of mitochondria in electron microscopy images with a generalist deep learning model trained on a diverse dataset. Cell Syst.

[15] W. Silversmith, “seung-lab/connected-components-3d: zenodo Release v1,” Sep. 2021, doi: 10.5281/ZENODO.5535251.

[16] L. Mescheder, M. Oechsle, M. Niemeyer, S. Nowozin, and A. Geiger, “Occupancy networks: learning 3D reconstruction in function space,” 2019.

[17] Xue F., Li J., Blu T. (Jun. 2017). Fast and accurate three-dimensional point spread function computation for fluorescence microscopy. JOSA A.

[18] Heusel M., Ramsauer H., Unterthiner T., Nessler B., Hochreiter S. (2017). GANs trained by a two time-scale update rule converge to a local nash equilibrium. Adv. Neural Inf. Process. Syst..

[19] Stucki N., Paetzold J.C., Shit S., Menze B.H., Bauer U. (2023). Topologically faithful image segmentation via induced matching of persistence barcodes. PMLR.

